# Comparison of FlowCam Macro and traditional microscopy for studying mesopelagic copepod community composition

**DOI:** 10.1093/plankt/fbag037

**Published:** 2026-06-26

**Authors:** Eloïse L-R Savineau, Kathryn B Cook, Anna Belcher, Sophie Fielding, Gabriele Stowasser, Geraint A Tarling, Daniel J Mayor

**Affiliations:** School of Ocean and Earth Science, University of Southampton, National Oceanography Centre, European Way, Southampton, SO14 3ZH, UK; National Oceanography Centre, Ocean Biogeoscience, European Way, Southampton SO14 3ZH, UK; Biosciences, Hatherly Building, University of Exeter, Exeter EX4 4PS, UK; National Oceanography Centre, Ocean Biogeoscience, European Way, Southampton SO14 3ZH, UK; Biosciences, Hatherly Building, University of Exeter, Exeter EX4 4PS, UK; British Antarctic Survey, Ecosystems team, High Cross, Madingley Road, Cambridge, CB3 0ET, UK; British Antarctic Survey, Ecosystems team, High Cross, Madingley Road, Cambridge, CB3 0ET, UK; British Antarctic Survey, Ecosystems team, High Cross, Madingley Road, Cambridge, CB3 0ET, UK; British Antarctic Survey, Ecosystems team, High Cross, Madingley Road, Cambridge, CB3 0ET, UK; National Oceanography Centre, Ocean Biogeoscience, European Way, Southampton SO14 3ZH, UK; Biosciences, Hatherly Building, University of Exeter, Exeter EX4 4PS, UK

**Keywords:** plankton imaging, zooplankton, stereomicroscopy

## Abstract

Semiautonomous imaging technologies are increasingly being used to characterize zooplankton communities, albeit with limited comparison to traditional techniques. Here we compared copepod community compositions obtained using a FlowCam Macro with those obtained using a stereomicroscope for the same set of samples. Broad-level community compositions obtained via the FlowCam Macro were similar to those using the microscope, although the microscope was able to achieve greater taxonomic resolution (e.g. genus/species level). Our data support the use of the FlowCam Macro as an alternative to time- and labour-intensive microscopy for broad-level taxonomic classification of mesozooplankton samples.

## INTRODUCTION

Recent years have seen a growing trend towards the digitalization of the ocean, with quantitative oceanographic imaging devices generating unprecedented volumes of data (Lombard *et al.*, 2019). Optical bench-top instruments, such as the FlowCam Macro (Yokogawa Fluid Imaging Technology), involving flow imaging cytometry, are now widely used to process and image zooplankton net samples, allowing for faster processing compared to traditional microscopic analysis (Detmer *et al.*, 2019; Cornils *et al*., 2022). This technology enables researchers to semiautonomously extract a wide range of information from imaged plankton, including morphological size, taxonomic identity and abundance. To fully capitalize on the potential of image-derived data, however, it is crucial to evaluate how this approach compares with conventional microscopic analyses. Here we use stereomicroscopy and the FlowCam Macro to compare copepod community composition, which dominates mesozooplankton composition (>70%) at all depths in the mesopelagic (Steinberg *et al.*, 2008; Stefanoudis *et al.*, 2019; Cook *et al.*, 2023).

## METHODS

Zooplankton samples were collected aboard the *RRS Discovery* during research cruise DY086 to the Scotia Sea in the Southern Ocean (12/11/2017–19/12/2017, 52.40 S, 40.06 W). A MOCNESS (Multiple Opening and Closing Net and Environmental Sampling System, 1 m^2^ rectangular opening, 330 μm mesh nets) was used to sample the mesozooplankton community at 8-depth-discrete intervals of 62.5 m, from 500 to 0 m and preserved in 4% borax-buffered formaldehyde (protocol in Cook *et al.*, 2023). Mesozooplankton copepod community composition was investigated for the 32 net samples using both stereomicroscopy and a FlowCam Macro. Stereomicroscopic identification and enumeration took place at the National Marine Fisheries Institute (NMFRI) Plankton Sorting and Identification Centre, Poland, following the standard protocol and a target enumeration >500 individuals per aliquot (Postel *et al.*, 2000). A target of 2000 particles per aliquot was imaged with the FlowCam Macro, following the standard protocol (Cook *et al.,* 2023). Due to high zooplankton biomass in the samples, both analysis methods involved subsampling aliquots (Table S1) for taxonomic analysis and enumeration, with abundances in the subsample being multiplied by the split fraction sampled to get the total abundances in the sample. Aliquot variability may introduce differences in community composition, such as via the “nugget effect” (Hatté *et al.,* 2026), where smaller aliquots potentially result in a less accurate representation of taxonomic diversity and rarer species in a subsample compared to larger aliquots. However, no relationship was found between the aliquot split fraction and the number of taxa identified (*F* = 1.58_1,62_, *P* = 0.214, *R*^2^ = 0.009). FlowCam Macro images were processed using Visual Spreadsheet software (version 4.3.55). Copepods were classified into nine broad taxonomic groups (Table S2). The copepod images obtained from the FlowCam Macro were mainly classified to order/family level (except for *Calanoides acutus* and *Rhincalanus gigas*, which were identified to species level), whereas the microscopic analysis identified to the genus and species levels (Table S2). To compare relative community compositions in a similar manner between the two methods, we reclassified the microscopic analysis to the same taxonomical level as the FlowCam Macro (detailed classification protocol in Table S2).

**Fig. 1:**
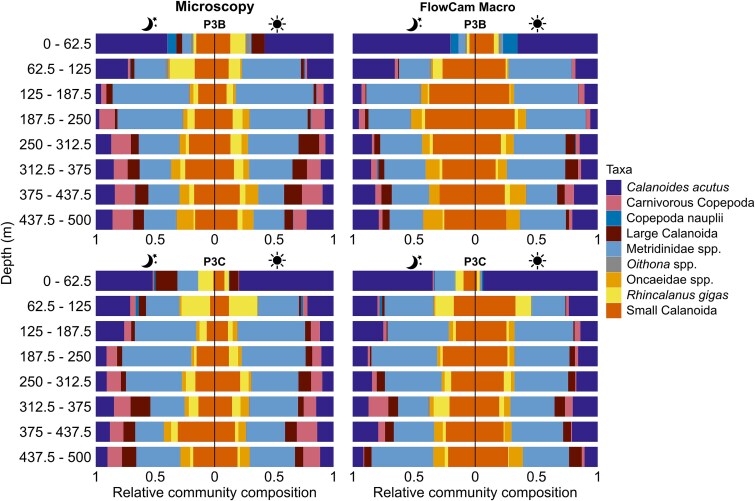
Vertical distribution of copepod taxonomic relative community compositions during visits P3B (top) and P3C (bottom) in the Scotia Sea analysed using microscopy (left) or the FlowCam Macro (right). Moon/Sun symbols represent night/day samples. Example images for each classification can be found in Table S2.

To assess differences in the copepod community composition between the two methods, permutational multivariate analysis of variance (PERMANOVA) and similarity percentage (SIMPER) analyses, both based on Bray–Curtis dissimilarities, were conducted using the vegan package version 2.6.4 (Oksanen *et al.*, 2022) in R version 4.2.3 (R Core Team, 2023). PERMANOVA tested the null hypothesis that the community compositions between the two methods do not differ, with a *P*-value < 0.05 rejecting the null hypothesis. Community composition data were expressed as a proportion of total abundance (i.e. relative community composition). To account for the fact that each sample was analysed using both methods, permutations were stratified (constrained) by sample ID. The significance of the test was evaluated using 999 permutations. Cumulative contributions were used to highlight the dominant taxa driving compositional differences. Two-dimensional nonmetric multidimensional scaling (NMDS) ordination plots were generated to visualize the similarities and differences in community composition between the two methods.

## RESULTS AND DISCUSSION

**Fig. 2:**
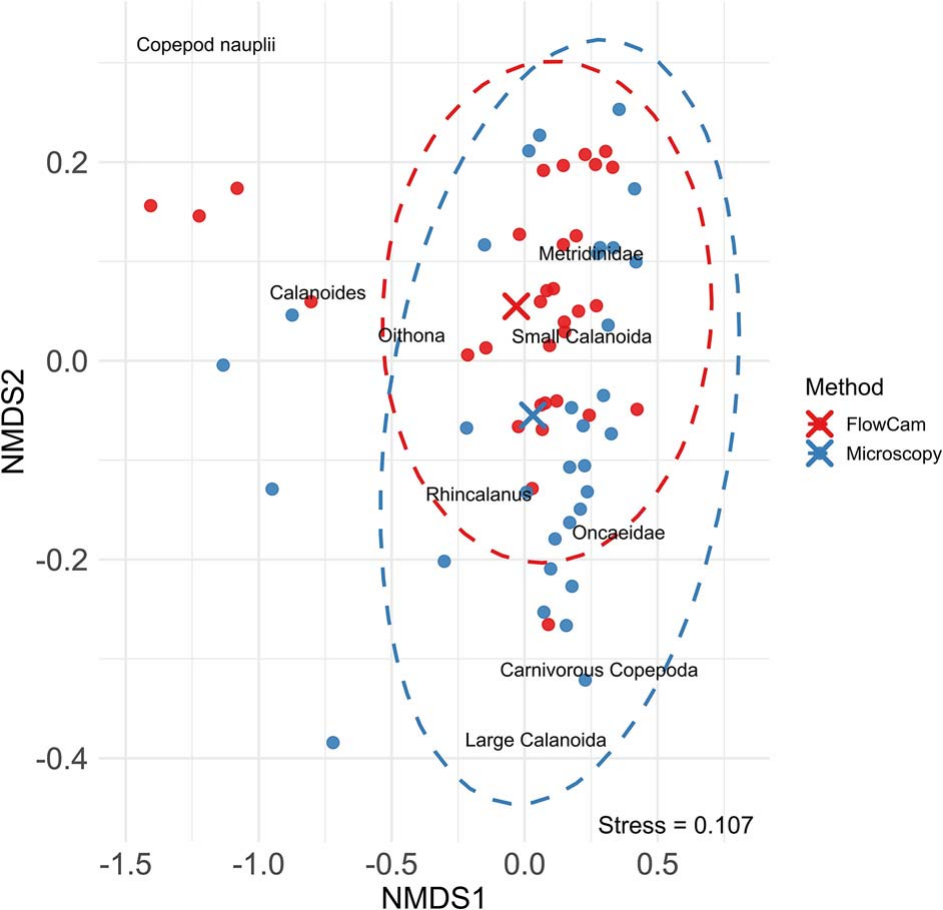
NMDS ordination of copepod relative community compositions based on Bray–Curtis dissimilarities*.* Each point represents a FlowCam or microscopy classified sample. Ellipses represent 95% confidence intervals around group centroids (centroids marked by “X”). Taxa labels indicate the position of individual taxa grouping in the ordination space. Calanoides = *Calanoides acutus*, Rhincalanus = *Rhincalanus gigas.*

The vertical distribution of copepod taxonomic relative community compositions is illustrated in Fig. 1. The FlowCam Macro classification clusters fell predominantly within the microscopy classification cluster (Fig. 2), forming partially overlapping groups, with the microscopy 95% confidence ellipse having a greater range.

Both the analysis method and depth were observed to significantly influence the copepod community composition (*F* = 20.2_1,63_, *P* = 0.001, *R*^2^ = 0.07 and *F* = 28.3_7,63_, *P* = 0.001, *R*^2^ = 0.72, respectively). The interactions between the two had no significant effect (*F* = 1.22_7,63_, *P* = 0.173, *R*^2^ = 0.03). Depth accounted for a greater proportion of the explained variance in copepod composition (72%) than method (7.4%), suggesting that while statistically significant, method-related compositional differences were minor relative to depth-driven variations. Moreover, although PERMANOVA detected a significant effect of analysis method, the absolute magnitude of composition difference was small, with mean Bray–Curtis dissimilarities between methods (0.357) only marginally greater than within-method dissimilarities (0.322), suggesting both methods captured highly similar community compositions. In contrast, depth showed greater between-group dissimilarity (0.357) relative to within-group dissimilarity (0.204), indicating depth was the primary driver of community structure. Overall, the two methods overlapped in community composition structure, with the two-dimensional NMDS solution having a stress value of 0.11, below the cut-off of 0.20, indicating good fit and representation of community dissimilarities/similarities (Fig. 2).

SIMPER analysis found that the main difference between the two methods arose from the FlowCam Macro having a greater contribution of small Calanoida and microscopy having a greater contribution of the carnivorous copepod *R. gigas* and large Calanoida (*P* < 0.01; Supplementary Table S3). We suggest these differences resulted from juvenile stages of species categorized as “Large Calanoida” by microscopy being classified in the “Small Calanoida” category of the FlowCam Macro from image-based size data. For example, all the stages (excluding nauplii) of *Calanus propinquus* were classified as “Large Calanoida” in the microscopy work; however, in the FlowCam Macro classification, unidentifiable calanoid copepods were classified as either small (<3000 μm) or large (>3000 μm) Calanoida; therefore, juvenile stages of large Calanoida could be classified as small Calanoida depending on their size. SIMPER analysis also found that *C. acutus*, Metridinidae and “Small Calanoida” were the primary contributors to dissimilarity between the surface (0–62.5 m) and deeper depths, accounting for 79% of cumulative dissimilarity.

Imaging technologies are increasingly being used due to their ability to couple size-based assessments with taxonomic knowledge of zooplankton communities. These data can be used to derive important ecological metrics, such as size spectra from which the slopes can be used as a proxy of trophic transfer efficiency through trophic levels (Tarling *et al.,* 2012). In terms of time efficiency, microscopy taxonomic and size-based measurement sample processing may take an experienced practitioner 8–16 hours, whereas processing via the FlowCam Macro takes an experienced user 1–2 hours, making the FlowCam Macro 8–16 times faster. Although the FlowCam Macro has an initial operational/investment cost, it makes up for it in time and labour costs. However, we suggest that the choice of imaging technologies is dependent on the research question at hand. When interested in broad-scale community composition and size structure, our results indicate that the FlowCam Macro may provide an effective alternative to time- and labour-intensive microscopy. In contrast, given the coarser taxonomic resolution of the FlowCam Macro compared to microscopy, if studies require genus- or species-level identification or stage- or sex-specific information then FlowCam Macro imaging may be less appropriate.

## CONCLUSION

This study provides a timely evaluation and quantitative evidence of the utility of the FlowCam Macro for broad-level taxonomic compositional analysis. Although imaging technologies can provide fast and reliable taxonomic and morphometric data, the lower taxonomic resolution of such devices does highlight the importance of retaining trained taxonomists.

## Supplementary Material

fbag037_Savineau_etal_JPR_supplementary_material_FINAL

## Data Availability

Data are available on request from the authors.
