# Supplementary material for: Comparison of FlowCam Macro and traditional microscopy for studying mesopelagic copepod community composition
Source: J Plankton Res. 2026 Jun 26;48(3):fbag037. doi: 10.1093/plankt/fbag037 (PMC13201259; doi:10.1093/plankt/fbag037)
Supplement: fbag037_Savineau_etal_JPR_supplementary_material_FINAL [file fbag037_savineau_etal_jpr_supplementary_material_final.docx]

**Comparison of FlowCam Macro and traditional microscopy for studying mesopelagic copepod community composition**

Eloïse L-R. Savineau^1,2,3^ *, Kathryn B. Cook^2,3^, Anna Belcher^4,5^, Sophie Fielding^4^, Gabriele Stowasser^4^, Geraint A. Tarling^4^, Daniel J. Mayor^2,3^

**Supplementary information**

Table SI. Description of each net sample and the aliquot sampled for each analysis method (FlowCam Macro or microscope). The same sample was analysed using each method.

| Sample ID | Station | Event | Net | Depth (m) | Time | Microscopy aliquot | FlowCam Macro aliquot |
| --- | --- | --- | --- | --- | --- | --- | --- |
| EV217_2 | P3B | 217 | 2 | 437.5-500 | Night | 1/8 | 1/16 |
| EV217_3 | P3B | 217 | 3 | 375-437.5 | Night | 1/4 | 1/16 |
| EV217_4 | P3B | 217 | 4 | 312.5-375 | Night | 1/8 | 1/16 |
| EV217_5 | P3B | 217 | 5 | 250-312.5 | Night | 1/16 | 1/16 |
| EV217_6 | P3B | 217 | 6 | 187.5-250 | Night | 1/16 | 1/16 |
| EV217_7 | P3B | 217 | 7 | 125-187.5 | Night | 1/16 | 1/16 |
| EV217_8 | P3B | 217 | 8 | 62.5-125 | Night | 1/32 | 1/64 |
| EV217_9 | P3B | 217 | 9 | 0-62.5 | Night | 1/64 | 1/96 |
| EV234_2 | P3B | 234 | 2 | 437.5-500 | Day | 1/4 | 1/16 |
| EV234_3 | P3B | 234 | 3 | 375-437.5 | Day | 1/4 | 1/16 |
| EV234_4 | P3B | 234 | 4 | 312.5-375 | Day | 1/8 | 1/16 |
| EV234_5 | P3B | 234 | 5 | 250-312.5 | Day | 1/8 | 1/16 |
| EV234_6 | P3B | 234 | 6 | 187.5-250 | Day | 1/32 | 1/16 |
| EV234_7 | P3B | 234 | 7 | 125-187.5 | Day | 1/32 | 1/32 |
| EV234_8 | P3B | 234 | 8 | 62.5-125 | Day | 1/128 | 1/32 |
| EV234_9 | P3B | 234 | 9 | 0-62.5 | Day | 1/128 | 1/16 |
| EV305_2 | P3C | 305 | 2 | 437.5-500 | Night | 1/4 | 1/16 |
| EV305_3 | P3C | 305 | 3 | 375-437.5 | Night | 1/8 | 1/16 |
| EV305_4 | P3C | 305 | 4 | 312.5-375 | Night | 1/4 | 1/16 |
| EV305_5 | P3C | 305 | 5 | 250-312.5 | Night | 1/16 | 1/16 |
| EV305_6 | P3C | 305 | 6 | 187.5-250 | Night | 1/32 | 1/16 |
| EV305_7 | P3C | 305 | 7 | 125-187.5 | Night | 1/16 | 1/16 |
| EV305_8 | P3C | 305 | 8 | 62.5-125 | Night | 1/32 | 1/16 |
| EV305_9 | P3C | 305 | 9 | 0-62.5 | Night | 1/256 | 1/64 |
| EV315_2 | P3C | 315 | 2 | 437.5-500 | Day | 1/8 | 1/16 |
| EV315_3 | P3C | 315 | 3 | 375-437.5 | Day | 1/8 | 1/16 |
| EV315_4 | P3C | 315 | 4 | 312.5-375 | Day | 1/8 | 1/16 |
| EV315_5 | P3C | 315 | 5 | 250-312.5 | Day | 1/16 | 1/16 |
| EV315_6 | P3C | 315 | 6 | 187.5-250 | Day | 1/32 | 1/16 |
| EV315_7 | P3C | 315 | 7 | 125-187.5 | Day | 1/32 | 1/16 |
| EV315_8 | P3C | 315 | 8 | 62.5-125 | Day | 1/32 | 1/16 |
| EV315_9 | P3C | 315 | 9 | 0-62.5 | Day | 1/512 | 1/64 |

Table SII. Description of each FlowCam Macro copepod classification group and the taxa representative of these groups based on the FlowCam Macro images and microscopic analysis. All FlowCam Macro images were obtained from samples from this study. Microscopy images were obtained from: ^1.^<https://inaturalist-open-data.s3.amazonaws.com/photos/67733907/medium.png>, ^2.^ [OceanPlankton](https://www.instagram.com/oceanplankton/) ^3.^<https://v3.boldsystems.org/index.php/Taxbrowser_Taxonpage?taxid=5780> Images are not to scale.

| Name of taxonomic group used to classify FlowCam Macro images | Typical taxa in the category based on microscopy work | Example Images | |
| --- | --- | --- | --- |
|  |  | Microscopy | FlowCam Macro |
| Oncaeidae | Oncaeidae spp.  *Triconia* spp.  *Oncaea* spp. | 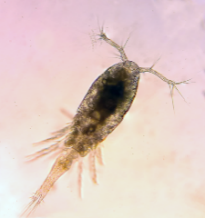  ^1^ | 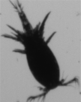 |
| *Oithona* spp. | *Oithona atlantica*  *Oithona similis*  *Oithona frigida*  *Oithona* spp. | 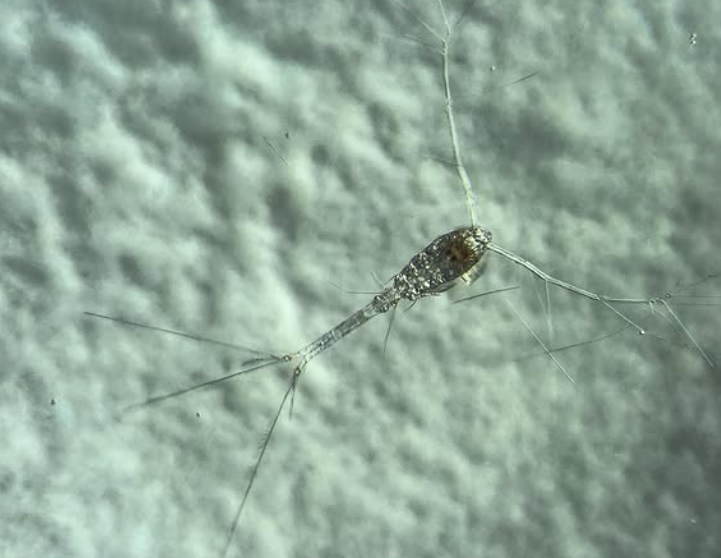  ^2^ | 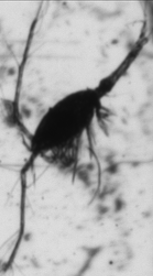 |
| Small Calanoida | Aetideidae (copepodites) *Aetideus armatus Aetideus australis* Calanoida spp. *Calocalanus* spp. *Clausocalanus* laticeps *Ctenocalanus* spp. *Racovitzanus antarcticus Scaphocalanus brevicornis Scaphocalanus farrani Scaphocalanus* spp. *Scolecithrichidae* spp. *Scolecithricella* spp. *Spinocalanus abyssalis Spinocalanus* spp. | 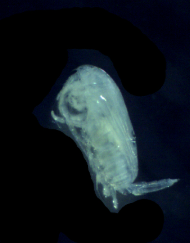  ^3^ | 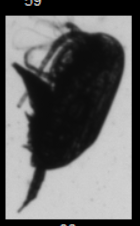 |
| Large Calanoida | *Amallothrix* spp. *Amallothrix valida Calanus propinquus Calanus simillimus Euchirella* spp*. Euaugaptilus* spp*. Gaetanus* spp*. Haloptilus* spp*. Lophothrix* spp*. Lucicutia* spp*. Neocalanus tonsus* Phaennidae | 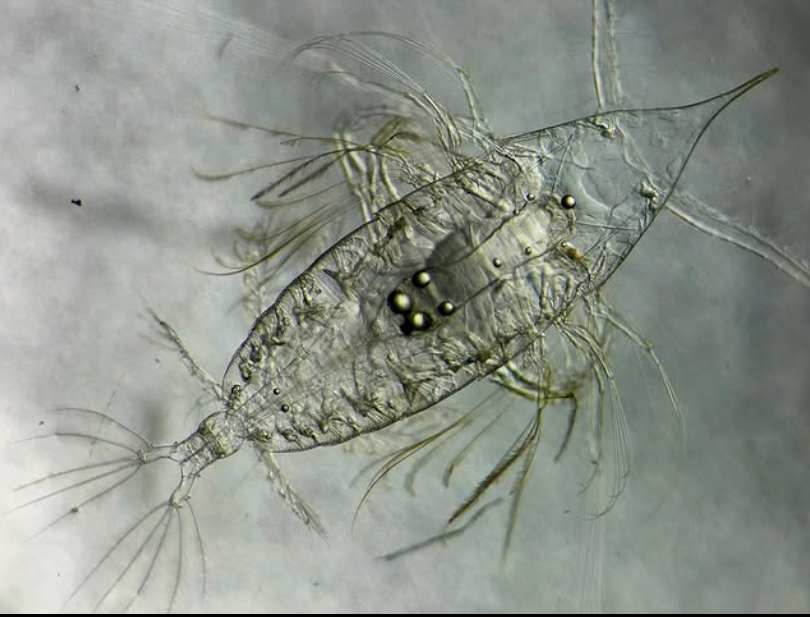  ^2^ | 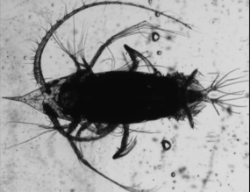 |
| *Calanoides acutus* | *Calanoides acutus* | 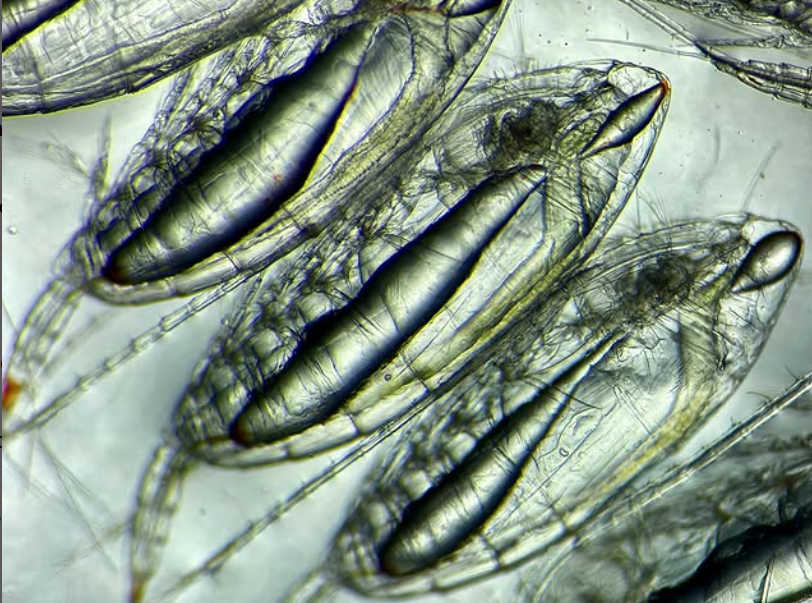  ^2^ | 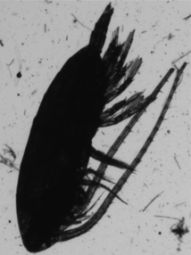 |
| Carnivorous Copepod | *Augaptilidae* spp*. Candacia* spp*. Candacia maxima* Euchaetidae spp*. Heterorhabdus* spp*. Heterorhabdus norvegicus Heterorhabdus spinifrons Paraeuchaeta* spp*. Paraeuchaeta antarctica Paraeuchaeta biloba Paraeuchaeta kurilensis* | 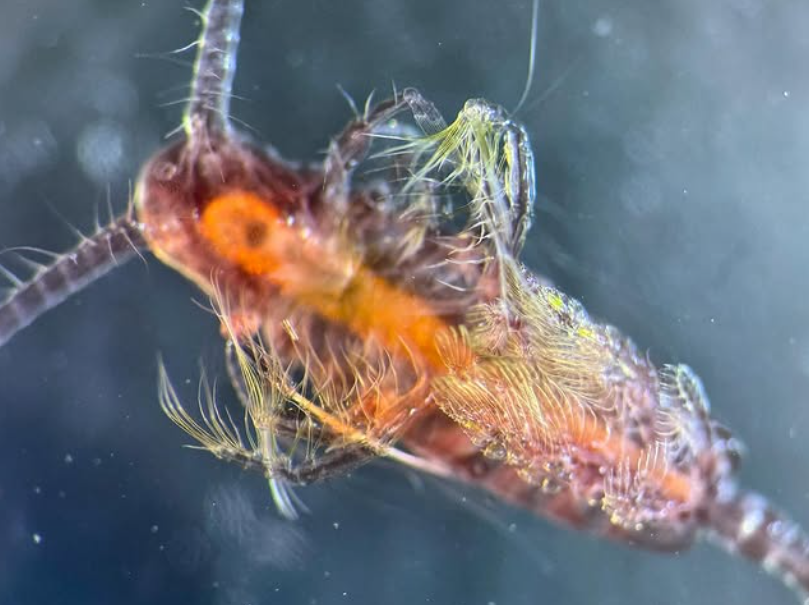  ^2^ | 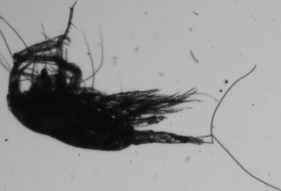 |
| *Rhincalanus gigas* | *Rhincalanus gigas* | 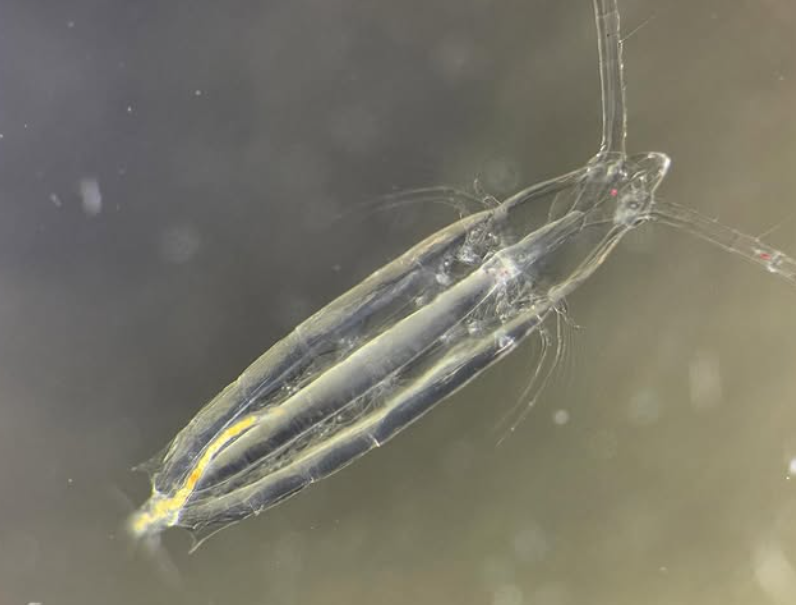  ^2^ | 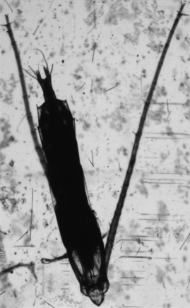 |
| Copepod nauplii (*Rhincalanus gigas)* | Copepod nauplii (not specified) | n/a | 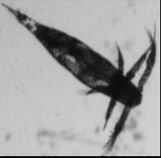 |
| Metridinidae | Metridinidae spp.  *Metridia gerlachei*  *Metridia lucens*  *Metridia curticauda*  *Metridia* spp*.*  *Pleuromamma antarctica* | 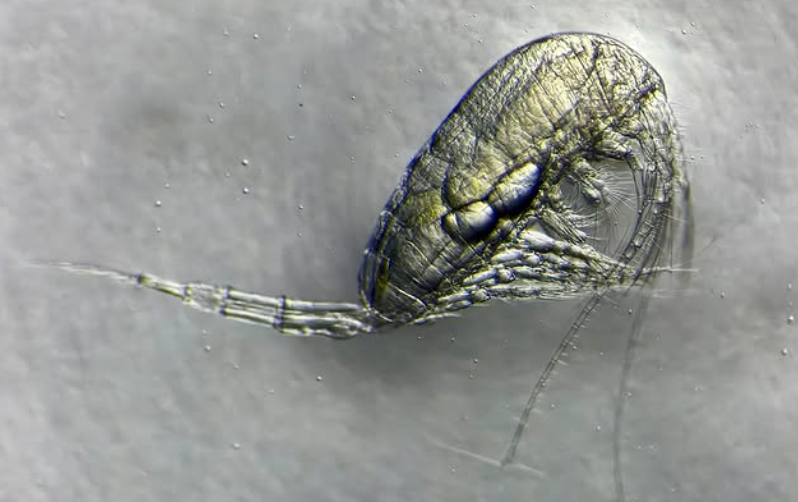  ^2^ | 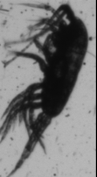 |

Table SIII. Summary of SIMPER (Similarity Percentages) analysis comparing copepod community composition between FlowCam Macro and microscopic methods of analysis. The table shows each taxon’s average contribution to the Bray–Curtis dissimilarity (Mean contribution), the standard deviation of the contribution (SD), mean relative community compositions in each group (FlowCam Macro, microscopy), cumulative contribution to total dissimilarity (Cumulative contribution), and the significance of each taxon’s contribution (p).

| Taxon | Mean contribution | SD | Mean FlowCam Macro | Mean microscopy | Cumulative contribution | P-value |
| --- | --- | --- | --- | --- | --- | --- |
| *Calanoides acutus* | 0.0926 | 0.1097 | 0.2167 | 0.1669 | 0.259 | 0.186 |
| Metridinidae | 0.0901 | 0.0650 | 0.3148 | 0.3035 | 0.511 | 0.534 |
| Small Calanoida | 0.0576 | 0.0390 | 0.2096 | 0.1286 | 0.672 | 0.001 |
| Carnivorous Copepod | 0.0330 | 0.0222 | 0.0289 | 0.0751 | 0.765 | 0.001 |
| *Rhincalanus gigas* | 0.0266 | 0.0265 | 0.0322 | 0.0616 | 0.839 | 0.002 |
| Large Calanoida | 0.0261 | 0.0198 | 0.0345 | 0.0657 | 0.912 | 0.001 |
| Oncaeidae | 0.0217 | 0.0170 | 0.0471 | 0.0372 | 0.973 | 0.364 |
| Copepod nauplii | 0.0059 | 0.0130 | 0.0072 | 0.0046 | 0.990 | 0.671 |
| *Oithona* spp. | 0.0037 | 0.0038 | 0.0052 | 0.0055 | 1.000 | 0.62 |
